# The Evolving Landscape of Anti-Clonal Therapy in Newly Diagnosed Systemic Light-Chain (AL) Amyloidosis: Evidence- and Time-Based Comparison with Multiple Myeloma

**DOI:** 10.3390/life16020363

**Published:** 2026-02-22

**Authors:** Rafael Ríos-Tamayo

**Affiliations:** 1 Department of Hematology, University Hospital Virgen de las Nieves, 18014 Granada, Spain; rriost33@gmail.com; 2 Instituto de Investigación Biosanitaria ibs.GRANADA, 18012 Granada, Spain; 3 CIBER Epidemiology and Public Health, 28029 Madrid, Spain

**Keywords:** systemic light-chain amyloidosis, multiple myeloma, treatment, anti-clonal therapy, immunotherapy, autologous stem cell transplant, anti-CD38 monoclonal antibodies, conjugated monoclonal antibodies, bispecific antibodies, chimeric antigen receptor T-cell therapy

## Abstract

Light-chain (AL) amyloidosis is a rare and incurable disease, classified under the category of plasma cell neoplasms and other diseases with paraproteins in the 5th Edition of the World Health Organization classification of lymphoid tumors. This entity shares some similarities with multiple myeloma (MM), remarkably a bone marrow infiltration of clonal plasma cells. Moreover, one out of five newly diagnosed cases of AL amyloidosis (NDAL) also fulfills the current diagnostic criteria for MM. A multidisciplinary therapy approach should be established, in which hematological therapy plays a crucial role. Anti-clonal therapy is the basis of hematological therapy, in addition to supportive therapy and emerging anti-fibrils therapy. In recent years, advances in the anti-clonal therapy for MM have progressively transferred to carefully selected patients with systemic AL amyloidosis, significantly improving outcomes in this rapidly changing field. This review aims to critically analyze the comparative evolution and evidence-based approach of anti-clonal therapy in NDAL vs. MM since the introduction of bortezomib. Participation in clinical trials remains the first option to consider in daily clinical practice.

## 1. Introduction

Immunoglobulin (Ig)-related light-chain (AL) amyloidosis is a challenging and heterogeneous entity included in the 5th Edition of the World Health Organization (WHO) classification of lymphoid tumors [1] in the category of “Plasma Cell Neoplasms and Other Diseases with Paraproteins”, and in the family of “Diseases with Monoclonal Ig Deposition”.

AL amyloidosis can be local, in about 5% of patients [2], or systemic, in most cases. Systemic involvement must always be excluded in cases of local disease at the time of diagnosis. The clinical behavior of true localized AL amyloidosis is commonly benign, with a very low risk of systemic transformation. Importantly, only systemic AL amyloidosis patients require systemic treatment.

Newly diagnosed (ND) systemic AL amyloidosis (NDAL) is characterized by the presence of a clonal population of Ig-secreting bone marrow plasma cells (cBMPCs), that produces a light chain (LC) as either an intact Ig or as LC-only monoclonal protein. This protein misfolds and forms insoluble amyloid fibrils that deposit in different organs, causing organ failure [3]. A correct diagnosis and AL typing is mandatory to exclude other types of amyloidosis with different therapeutic approaches. The spectrum of monoclonal gammopathies (MGs) includes MG of uncertain significance (MGUS), MG of clinical significance (MGCS), multiple myeloma (MM), Waldenström macroglobulinemia (WM), and other entities [4]. The bone marrow aspirate and biopsy are crucial to characterize cBMPCs and confirm a timely and accurate diagnosis [5].

The prognostic impact of comorbidity in MM and systemic AL amyloidosis is well documented [6,7]. About 20% of patients with systemic AL amyloidosis fulfill the current diagnostic criteria for MM (AL/MM) [2], representing a peculiar form of comorbidity with negative prognostic impact. Nonetheless, AL amyloidosis may be associated with other MGs such as MGUS; MGCS, particularly MG of renal significance; WM; other types of non-Hodgkin lymphomas; and the full spectrum of MM, ranging from smoldering MM (SMM) to plasma cell leukemia.

MM and systemic AL amyloidosis are both complex, incurable, and heterogeneous diseases. However, the epidemiology of these two entities [8,9] presents key differences, mainly in terms of incidence and prevalence, with systemic AL amyloidosis considered as a rare disease. Consequently, the pace of research has been agile, thrilling, and dizzying in MM, while the rhythm of research in AL amyloidosis has been slow and challenging. Interestingly, AL/MM patients have been able to benefit from recently approved therapeutic advances in MM, showing deeper hematological and cardiac responses. The response dynamics studies demonstrated that achieving an early and deep hematological response was necessary, in most cases, to reach deep and long-lasting cardiac response, which is the crucial endpoint in terms of overall survival (OS) [10,11]. This has been confirmed mainly in the setting of observational real-world studies. Unfortunately, patients with both diseases have usually been mutually excluded from specific clinical trials for one of the two entities.

The use of T-cell redirecting immunotherapy has transformed the treatment of relapsed/refractory (RR) MM (RRMM). Both chimeric antigen receptor (CAR) T cells and T-cell engagers have changed this clinical scenario, and they are being investigated in ND MM (NDMM). The prognostic relevance of achieving and maintaining measurable residual disease (MRD) negativity (MRD^−^) has been confirmed in both NDMM and RRMM [12,13], becoming a surrogate early endpoint of progression-free survival (PFS) and OS. The growing evidence regarding the role of MRD in systemic AL amyloidosis seems to point in the same direction as that in the case of MM [14].

The current therapeutic approach of systemic AL amyloidosis should be necessarily personalized, comprehensive, and multidisciplinary [15,16], involving a group of specialists integrated into a specific AL clinical unit. Hematological treatment should be addressed at three levels, i.e., first, supportive therapy [17]; second, anti-clonal therapy [18,19]; and third, anti-fibrils therapy (currently only available in clinical trials) [20].

Epidemiology can help to unveil clinical disparities, and therefore, it could be considered the first level of heterogeneity. The incidence and prevalence of AL are much lower than those of MM, justifying slower research development.

Despite similarities between AL and MM (cBMPCs infiltration and similar anti-clonal therapy), a time-based comparative approach in the evolution of the anti-clonal therapies is lacking. This narrative review aims to comparatively explore the evolving bortezomib-based anti-clonal therapy in NDAL vs. MM, focusing on the respective accumulated evidence and the speed with which it was obtained.

## 2. The Evolution of Anti-Clonal Therapy in Systemic AL Amyloidosis over Time

The history of anti-clonal therapy in systemic AL amyloidosis mirrors what happened in MM, with some peculiarities, nuances, and a certain delay. Overall, three periods could be pointed out.

### 2.1. Second Half of the 20th Century, the Chemotherapy Era

The history of systemic AL amyloidosis has been recently summarized. Remarkably, more than a century passed from the first clinical use of the term “amyloid” by Rudolf Virchow (1854) and the first description of a patient with primary amyloidosis, attributed to Samuel Wilks (1856), to the use of alkylating agents in the 1960s [21]. Melphalan (M) was first used in MM [22], and later in AL amyloidosis, when the close relationship between AL and MM was pointed out [23,24,25].

M was the most frequently used during the past century in both NDAL and RRAL. However, a shift to cyclophosphamide (C) occurred over the past two decades due to its immunomodulatory effect and a better safety profile in comparison with the results for M [26]. Since 2005, bendamustine (B) has been an option, particularly for RRAL [27].

Alkylating agents are usually administered in combination with corticosteroids, mainly prednisone (p) or dexamethasone (d).

The seemingly outdated chemo era has reached the present day. C remains in use, in combination with daratumumab (D), bortezomib (V), and dexamethasone, (d), as a quadruplet (DVCd), being the current standard of care (soc) for NDAL patients [18,19]. On the other hand, high-dose M (HDM) is still the preferred conditioning regimen for autologous stem cell transplant (ASCT) in transplant-eligible AL patients [28].

### 2.2. First Two Decades of the 21st Century, the Era of the New Agents

Immunomodulatory drugs (IMiDs) and proteasome inhibitors (PIs), the so-called new agents, have gained special prominence in the treatment of AL patients over the last two decades.

Regarding IMiDs [29], thalidomide (T) was the first-in-class drug used in this setting, followed by lenalidomide (R) and pomalidomide (P). IMiDs are mainly used in RRAL patients. These all-oral drugs should be used with caution, particularly in patients with cardiac involvement, due to their toxicity profile.

PIs [30] have been a cornerstone in the treatment of AL patients over the current century, following their success in treating MM. V is still the PI most frequently used, remaining involved in the present soc for NDAL. Intravenous carfilzomib and oral ixazomib are other less-frequently used PIs.

Both families of drugs can eventually be combined with each other, with alkylators, and with corticosteroids, resulting in different and well-known combinations such as VCd [31].

### 2.3. Third Decade of the 21st Century, the Immunotherapy Era

The recognition of specific therapeutic targets in cBMPCs allowed for the development of monoclonal antibodies (mAbs) [32] and Ab–drug conjugates (ADC) [33], deeply changing the treatment paradigm of PC disorders, first in MM and then in AL.

The emergence of antiCD38 mAbs represented a revolution in the treatment of AL, with D being the first antiCD38 used [34]. The addition of D to VCd in the phase 3 ANDROMEDA trial [35] was the basis for the first FDA approved regimen for NDAL in 2021. Moreover, on 19 November 2025, the FDA granted traditional approval to daratumumab and hyaluronidase-fihj (Darzalex Faspro) in combination with VCd, based on the final analysis of the ANDROMEDA trial. Subsequently, other anti-CD38 mAbs were developed. Isatuximab (Isa) as monotherapy has demonstrated similar results to those for D in the RRAL setting [36].

Elotuzumab (Elo) is a mAb targeting the signaling lymphocytic activation molecule family member F7 (SLAMF7). It has been mainly used in RRAL, in combination with IMiDs [37].

B-cell maturation antigen (BCMA) is a glycoprotein expressed on cBMPCs, which has become a key therapeutic target for MM and AL. Several BCMA-targeting approaches have been developed [38,39]. First, belantamab mafodotin (belamab) is a BCMA-directed IgG1 conjugated to monomethyl auristatin F, a microtubule-disrupting agent. Curiously, the first report on the use of belamab as a single agent in AL was also in 2021, involving six RR AL/MM patients [40]. Second, T-cell redirecting bispecific Abs (BsAbs) comprise another treatment approach. Several BCMA-CD3 BsAbs have been used in RRAL, mainly teclistamab [41] and elranatamab [42], whereas ABBV-383 (etentamig) [43], linvoseltamab, and others are currently under development. Third, chimeric antigen receptor (CAR) T-cells [44] are also used. Several anti-BCMA CAR-T cells have been used in AL: NXC201 (HBI0101) [45,46], idecabtagene vicleucel (ide-cel, BB2121) [47], ciltacabtagene autoleucel (cilta-cel) [48,49], and cesnicabtagene autoleucel (ARI0002h) [50]. Fourth, there are also emerging anti-BCMA therapeutic approaches. Trispecific Abs (TsAbs) may potentially overcome acquired resistance to BsAbs. JNJ-5322 is a TsAb dually targeting BCMA and G protein-coupled receptor class C group 5 member D (GPRC5D), showing a 100% overall response rate (ORR) in a recent phase 1 study (NCT05652335) for RRMM patients [51].

Despite advances in anti-clonal therapy, the hope placed in anti-fibrils therapy has yet to materialize. The phase 3 AFFIRM-AL trial (NCT04973137) of birtamimab was discontinued in May 2025 after it failed to meet its primary endpoint of time to all-cause mortality. Similarly, CAEL-101 (anselamimab) did not meet its primary endpoint in the CARES program. These results underscore the ongoing challenge of amyloid clearance and the continued reliance on deep hematologic response.

## 3. The Evolving Landscape of Bortezomib-Based Therapies in NDAL vs. NDMM

New drugs for MM and AL are usually first tested in the RR setting and then in ND patients. V has demonstrated a crucial role in both entities since its accelerated approval by the FDA in 2003 for its use in RRMM, based on the phase II SUMMIT trial [52]. Five years passed before its approval for NDMM in 2008.

From the beginning, V was commonly used in combination with d (Vd), due to a well demonstrated synergism. Step by step, different Vd-based combinations that were used in NDMM were also tested in NDAL, except for those associated with an unacceptable toxicity profile, particularly in terms of cardiotoxicity, such as Adriamycin (A) in Vad (bortezomib, adriamycin, dexamethasone). Progressively, different alkylating agents were associated with the Vd backbone, mainly M, C, and B, as well as IMiDs (T, R, P) and mAbs (D, Isa, Elo), in diverse triplets or quadruplets.

AL is a rare entity, and therefore, the evidence in the evolving landscape of the treatment in NDAL patients comes mainly from case reports and small single-center series of real-world retrospective studies. To avoid potential bias in the comparison between the respective evolution of NDMM and NDAL anti-clonal therapy, only chronologically ordered, V-based, phase II and III clinical trials for both clinical scenarios were analyzed in Table 1, after an intensive English PubMed search including the following terms: “multiple myeloma, systemic AL amyloidosis, clinical trials, induction, therapy, treatment, bortezomib, and newly diagnosed”.

Several phase II and two phase III trials [53,55,57,59,61,62] assessed Vd in NDMM (2005–2015), whereas in the NDAL setting, a small observational study [110] including 18 consecutive AL patients (11 NDAL and 7 RRAL) treated with Vd pointed out an excellent 94% ORR. Shortly after, a phase III trial tested direct ASCT vs. two cycles of Vd as the induction, followed by ASCT [54], showing a better outcome for patients treated with Vd induction. The corresponding phase II studies for NDAL [56,58,60] presented an evident delay (2015–2020) with respect to those for NDMM.

VAd was utilized as the induction in NDMM mainly during the first decade of the current century [63,64,65], but this triplet was not applied to NDAL due to the A-associated cardiotoxicity risk.

Until the advent of D, the soc for NDAL patients was a combination of Vd and an alkylating agent, M (VMd or VMp) or C (VCd). Again, a huge difference in the time frame and the associated supporting evidence was obvious between both clinical scenarios. Regarding VMp, several phase II and III trials (2008–2025) demonstrated excellent outcomes in transplant-ineligible NDMM [66,68,69,70,71]. Remarkably, 682 patients were recruited in the phase III VISTA trial (2008) over less than two years [66]. In contrast, only one relatively recent (2020) phase III trial established VMd as the soc for NDAL [67]. This study included 109 patients, and the recruitment took place over five years.

The clinical impact of VCd was also extensively analyzed in phase II and III trials (2009–2019) for NDMM patients [72,74,75,76,77,78,79], whereas most studies investigating the role of this triplet in NDAL patients were retrospective. Only one recent trial [73] demonstrated a lack of clinical benefit when doxycycline was added to VCd compared with the use of VCd alone in cardiac NDAL patients. The most relevant retrospective study using VCd in NDAL enrolled 230 patients over more than six years [111].

Frontline BVp was explored in NDMM [80,81], but no trials were identified involving the use of this triplet in NDAL patients.

Regarding combinations of V with IMiDs, several trials were identified for NDMM patients with the following triplets: VTd [82,83,84], VRd [85,86,87,88], and PVd [89]. However, no trials with these triplets were conducted in NDAL patients.

As expected, all the previously mentioned anti-clonal combinations were challenged with the progressive introduction of different mAbs directed against specific therapeutic targets such as anti-SLAMF7 (Elo), anti-CD38 (D, Isa), and anti-BCMA (Belamaf). Overall, VRd has been the most widely used regimen to combine with the new mAbs in NDMM. In these instances, EloVRd registered excellent results in NDMM [90,91,92], but trials are lacking in NDAL.

Following thrilling clinical development schedules, first D- and then Isa-based anti-clonal therapies have highlighted the key role of targeted anti-CD38 therapy in NDMM and NDAL. D was the first anti-CD38 used in association with the full range of previous V-based regimens in NDMM. Interestingly, two phase II trials regarding the use of DVd in MM [93] and in AL [94] were published almost simultaneously. The DVMp regimen was successfully applied to transplant-ineligible (TI) NDMM [95], but no trials were developed for NDAL. Remarkably, DVCd was also tested earlier in NDMM [96] than in NDAL [35], and other recent trials have confirmed good outcomes in both settings [97,98,99,100]. Regarding D-based combinations with IMiDs, DVTd [101], and mainly DVRd [102,103,104,105], which is the current soc, have been extensively employed in NDMM, but no trials with these combinations have been tested in NDAL, probably due to the toxicity profile of IMiDs.

Alternatively, Isa-based combinations, mostly IsaVRd [106,107,108,109], have also been developed for NDMM with excellent results, but no completed trials remain available for NDAL.

New immunotherapy approaches in NDAL are being evaluated in ongoing clinical trials, as well as anti-clonal therapy in the RRAL setting, but they are beyond the scope of this review. 

## 4. Discussion

The global landscape of V-based phase II/III trials focused on NDMM and NDAL over the last two decades is summarized in Table 2.

A total of 58 trials (27 phase III) were included in the analysis, and only 10 of them (17.2%) were performed in the setting of NDAL.

A total of 23 (85.2%) out of 27 phase III trials were performed in NDMM, and only 4 in NDAL [35,54,67,100], associated with Vd, VMd, and DVCd.

A total of 16 combinations, including doublets, triplets, and quads, were compared in both scenarios. There was at least one NDAL study to compare in five (31.2%) of these regimens (Vd, VMp, VCd, DVd, and DVCd), highlighting the real evolution of anti-clonal therapy in NDAL in which V and D appear as the crucial drugs, along with an increasingly cautious use of corticosteroids.

Regarding the long-lasting debate about the use of alkylators in both scenarios, HDM conditioning with ASCT remains in use for transplant-eligible NDMM and NDAL patients. On the other hand, C is the preferred alkylator for induction in NDAL (DVCd).

Overall, the outcome of AL patients is improving over time, but the prognosis remains poor in a subgroup of these. Staging plays a critical role in the evolving prognostic evaluation in this setting. A newly validated AL International Staging System (AL-ISS) defines an “ultra-poor risk” group (Stage IIIC) with a median OS of approximately 7 months, even in the current D-based era [112]. This group is defined by NT-proBNP ≥ 8500 ng/L, high-sensitivity Troponin T ≥ 50 ng/L, and a Global Longitudinal Strain ≥ -9%, and should be managed with tailored risk-adapted therapy.

**Figure 1:**
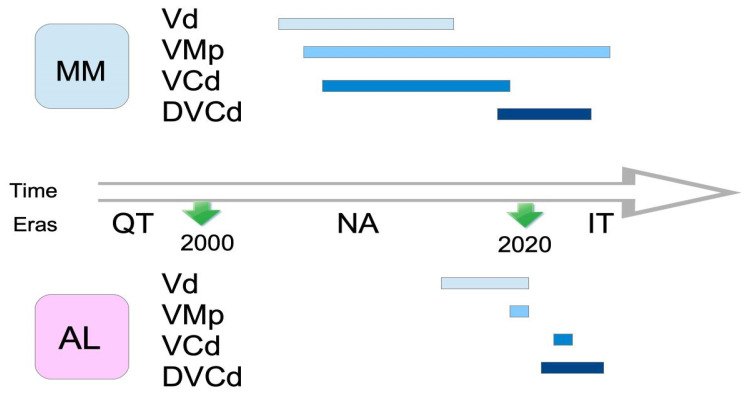
Comparative delay in key bortezomib-based phase II and III trials in newly diagnosed multiple myeloma vs. newly diagnosed systemic light-chain (AL) amyloidosis. C: cyclophosphamide, D: daratumumab, d: dexamethasone, IT: immunotherapy era, M: melphalan, NA: new agents’ era, QT: chemotherapy era, and V: bortezomib.

Figure 1 graphically shows the significant delay in phase II and III trials between NDMM and NDAL regarding four common V-based regimens.

## 5. Conclusions

The implementation of V-based phase II and III trials in NDAL shows a significant delay when compared with that for NDMM over the last two decades. Moreover, the number of trials, particularly phase III trials, is significantly lower in the NDAL setting. This is mainly attributed to differences in the epidemiological background of both entities, underlining the difficulty of performing clinical trials in the context of very rare diseases. Despite this, large collaborative research platforms, led by referral centers, increasingly allow studies to be carried out.

At the present time, two quads (DVRd and DVCd), differing in the use of only one drug, are the soc for NDMM and NDAL, respectively. Remarkably, since its introduction two decades ago, V remains a cornerstone in the induction of both entities. On the other hand, anti-CD38 mAbs have also demonstrated a crucial role in the induction therapy of these two scenarios, as well as in the event of the coexistence of both diseases (AL/MM).

Hopefully, the research background for the introduction of new therapeutic advances in NDAL will be shortened in the coming years and will resemble the process for NDMM, a model of highly efficient dynamic research.

## Figures and Tables

**Table 1:** Comparative evolution of bortezomib-based phase II/III trials in newly diagnosed multiple myeloma vs. newly diagnosed light-chain (AL) amyloidosis.

Anti-Clonal Therapy	NDMM				NDAL	
	Year/ Authors	Study	*n*	Name	Year/ Author	Study
**Vd**	2005 Jagannath, S. et al. [53]	Phase II	32	-	2014 Huang, X. et al. [54]	Phase III Vdx2 & ASCT/ASCT
	2006 Harousseau, J.L. et al. [55]	Phase II	48	-	2015 Sanchorawala, V. et al. [56]	Phase II
	2007 Rosiñol, L. et al. [57]	Phase II	40	-	2019 Minnema, M.C. et al. [58]	Phase II
	2010 Harousseau, J.L. et al. [59]	Phase III Vd/vAd	121/121	IFM 2005-01	2020 Landau, H. et al. [60]	Phase II
	2015 Girnius, S.K. et al. [61]	Phase II	50	NCT01090921	-	
	2015 Niesvizky, R. et al. [62]	Phase III Vd/VTd/VMp	168/167/167	UPFRONT NCT00507416	-	
**VAd**	2009 Jakubowiak, A.J. et al. [63]	Phase II	40	-	-	
	2010 Palumbo, A. et al. [64]	Phase II	102	-	-	
	2012 Sonneveld, P. et al. [65]	Phase III VAd/vAd	413/414	HOVON-65/ GMMG-HD4	-	
**VMp/VMd**	2008 San Miguel, J.F. et al. [66]	Phase III VMp/Mp	344/338	VISTA NCT00111319	2020 Kastritis, E. et al. [67]	Phase III VMd/Md
	2010 Mateos, M.V. et al. [68]	Phase III VMp/VTp	130/130	GEM05 NCT00443235	-	
	2010 Palumbo, A. et al. [69]	Phase III VMp/VMpT	257/254	GIMEMA-MM-03-05 NCT01063179	-	
	2016 Mateos, M.V. et al. [70]	Phase II VMp/Rd seq vs. alt	118/115	GEM2010 NCT01237249	-	
	2025 Mateos, M.V. et al. [71]	Phase III VMp-Rd/KRd/ DKRd	154/154/153	GEM-2017FIT NCT03742297	-	
**VCd**	2009 Reeder, C.B. et al. [72]	Phase II	33	-	2022 Shen, K.-n et al. [73]	NR VCd/VCddox
	2010 Bensinger, W.I. et al. [74]	Phase II VCd /VTd seq	44	-	-	
	2012 Kumar, S. et al. [75]	Phase II VCd/VCd mod /VRd/VRCd	33/17/42/48	EVOLUTION NCT00507442	-	
	2015 Mai, E.K. et al. [76]	Phase III VCd/VAd	251/251	MM5-GMMG	-	
	2016 Moreau, P. et al. [77]	Phase III VCd/VTd	170/170	IFM 2013-14 NCT01564537	-	
	2017 Einsele, H. et al. [78]	Phase II	414	DSMM XI NCT00833560	-	
	2019 Tanaka et al. [79]	Phase II	38	-	-	
**BVp**	2013 Berdeja, J. et al. [80]	Phase II	43	-	-	
	2015 Mateos, M.V. et al. [81]	Phase II	60	NCT01376401	-	
**VTd**	2010 Cavo, M. et al. [82]	Phase III VTd/Td	241/239	NCT01134484	-	
	2012 Rosiñol, L. et al. [83]	Phase III VTd/Td/QT + V	56/56/57	NCT00461747	-	
	2015 Ludwig, H. et al. [84]	Phase II VTd/VTCd	49/49	NCT00531453	-	
**VRd**	2017 Durie, B.G.M. et al. [85]	Phase III VRd/Rd	235/225	SWOG S0777 NCT00644228	-	
	2019 Rosiñol, L. et al. [86]	Phase III	458	GEM2012 NCT01916252	-	
	2020 Kumar, S. et al. [87]	Phase III VRd/KRd	542/545	ENDURANCE NCT01863550	-	
	2024 Ailawadhi, S. et al. [88]	Phase III VRd/DRd	459	SWOG S2209 NCT05561387	-	
**PVd**	2023 Saj, F. et al. [89]	Phase II	34	POMACE	-	
**EloVRd**	2017 Laubach, J. et al. [90]	Phase II	41	NCT02375555	-	
	2021 Usmani, S.Z. et al. [91]	Phase II EloVRd/VRd	48/52	SWOG-1211 NCT01668719	-	
	2024 Mai, E.K. et al. [92]	Phase III EloVRd/VRd	279/280	GMMG-HD6 NCT02495922	-	
**DVd**	2023 Nagarajan, C. et al. [93]	Phase II	27	AMN006 NCT03695744	2024 Shen, K.-n et al. [94]	Phase II
**DVMp**	2018 Mateos, M.V. et al. [95]	Phase III DVMp/VMp	350/356	ALCYONE NCT02195479	-	
**DVCd**	2019 Yimer et al. [96]	Phase II	86	LYRA NCT02951819	2021 Kastritis, E. et al. [35]	Phase III DVCd/VCd
	2023 Beksac, M. et al. [97]	Phase II	29	EMN19 NCT04166565	2023 Rosenzweig, M. et al. [98]	Phase II DVCd
	2024 Mollee, P. et al. [99]	Phase II DVCd/VCd	64/57	AMaRC 03-16 ACTRN12617000202369	2024 Hagen, P. et al. [100]	Phase III DVCd/DVCd-ASCT
**DVTd**	2020 Roussel, M. et al. [101]	Phase III DVTd/VTd	543/542	CASSIOPEIA NCT02541383	-	
**DVRd**	2023 Voorhees, P.M. et al. [102]	Phase II DVRd/VRd	104/103	GRIFFIN NCT02874742	-	
	2024 Sonneveld, P. et al. [103]	Phase III DVRd/VRd	355/354	PERSEUS NCT03710603	-	
	2025 Usmani, S.Z. et al. [104]	Phase III DVRd/VRd	197/198	CEPHEUS NCT03652064	-	
**DCVRd**	2023 Kaiser, M.T. et al. [105]	Phase II	107	OPTIMUM (MUKnine) NCT03188172	-	
**IsaVRd**	2022 Goldschmidt, H. et al. [106]	Phase III IsaVRd/VRd	331/329	GMMG-HD7 NCT03617731	-	
	2024 Facon, T. et al. [107]	Phase III IsaVRd/VRd	265/181	IMROZ NCT03319667	-	
	2024 Leleu, X. et al. [108]	Phase III IsaVRd/IsaRd	135/135	BENEFIT NCT04751877	-	
	2025 Askeland, F.B. et al. [109]	Phase II IsaVRd	51	REST NCT04939844	-	

A: pegylated liposomal doxorubicin (adriamycin), alt: alternating, B: bendamustine, C: cyclophosphamide, D: daratumumab, d: dexamethasone, dox: doxycycline, Elo: elotuzumab, Isa: istuximab, K: carfilzomib, M: melphalan, mod: modified, NR: not reported, P: pomalidomide, p: prednisone, QT: chemotherapy, R: lenalidomide, seq: sequential, T: thalidomide, V: bortezomib, and v: vincristine.

**Table 2:** Summary of bortezomib-based phase II/III trials regarding newly diagnosed multiple myeloma vs. systemic light-chain (AL) amyloidosis.

Regimen	*n* (MM/AL)	Period MM	Period AL
Vd	6/4	2005–2015	2014–2020
VAd	3/0	2009–2012	-
VMp/VMd	5/1	2008–2025	2020
VCd	7/1	2009–2019	2022
BVp	2/0	2013–2015	-
VTd	3/0	2010–2015	-
VRd	4/0	2017–2024	-
PVd	1/0	2023	-
EloVRd	3/0	2017–2024	-
DVd	1/1	2023	2024
DVMp	1/0	2018	-
DVCd	3/3	2019–2024	2021–2024
DVTd	1/0	2020	-
DVRd	3/0	2023–2025	-
DCVRd	1/0	2023	-
IsaVRd	4/0	2022–2025	-
Total	48/10	2005–2025	2014–2024

A: pegylated liposomal doxorubicin (adriamycin), B: bendamustine, C: cyclophosphamide, D: daratumumab, d: dexamethasone, Elo: elotuzumab, Isa: istuximab, M: melphalan, mod: modified, NR: not reported, P: pomalidomide, p: prednisone, R: lenalidomide, T: thalidomide, V: bortezomib.

## Data Availability

Not applicable.

## Abbreviations

The following abbreviations are used in this manuscript:

ADC	antibody-drug-conjugates
AL	light-chain amyloidosis
AL/MM	light-chain amyloidosis and multiple myeloma
ASCT	autologous stem cell transplant
B	bendamustine
BCMA	B-cell maturation antigen
Belamaf	belantamab mafodotin
BsAbs	bispecific antibodies
C	cyclophosphamide
CAR	chimeric antigen receptor
cBMPCs	clonal bone marrow plasma cells
d	dexamethasone
D	daratumumab
Elo	elotuzumab
HDM	high-dose melphalan
Ig	immunoglobulin
IMiDs	immunomodulatory drugs
Isa	isatuximab
LC	light chain
M	melphalan
mAbs	monoclonal antibodies
MGs	monoclonal gammopathies
MGCS	monoclonal gammopathy of clinical significance
MGUS	monoclonal gammopathy of uncertain significance
MM	multiple myeloma
MRD	measurable residual disease
ND	newly diagnosed
NDAL	newly diagnosed systemic AL amyloidosis
NDMM	newly diagnosed multiple myeloma
ORR	overall response rate
OS	overall survival
p	prednisone
P	pomalidomide
PIs	proteasome inhibitors
R	lenalidomide
RR	relapsed/refractory
SMM	smoldering multiple myeloma
T	thalidomide
TsAbs	trispecific antibodies
V	bortezomib
WM	Waldenström macroglobulinemia
